# Rhodamine B/gold nanoparticles-coloaded UiO-66 as a novel probe for highly sensitive and dual-mode detection of Potato virus S in field samples

**DOI:** 10.3389/fmicb.2026.1876426

**Published:** 2026-06-17

**Authors:** Xiangting Li, Meng Ren, Shuhang Deng, Lingli Lei, Ping Ma, Yingshuai Liu

**Affiliations:** 1 School of Materials and Energy, Southwest University, Chongqing, China; 2 School of Tourism & Urban-Rural Planning, Xichang University, Xichang, China; 3 Hubei Key Laboratory of Environmental Risks and Related Diseases Precision Control, Hubei University of Science and Technology, Xianning, China; 4 Key Laboratory of Structure-Specific Small Molecule Drugs at Chengdu Medical College of Sichuan Province, School of Pharmacy, Chengdu Medical College, Chengdu, China

**Keywords:** dual-signal, lateral-flow immunoassay (LFIA), metal-organic framework, on-site detection, Potato virus S

## Abstract

**Introduction:**

Potato virus S (PVS) causes severe damage to Solanaceae plants, especially potato, due to its widespread infection and the reliance on laboratory-based reverse transcription-polymerase chain reaction (RT-PCR) testing. This work intends to develop a rapid, highly sensitive, and dual-signal platform for on-site PVS detection.

**Methods:**

We developed a highly sensitive lateral-flow immunoassay (LFIA) capable of colorimetric and fluorescent dual-mode detection based on a novel core-shell structured probe, which consists of a porous metal-organic framework UiO-66 encapsulating rhodamine B inside and loading gold nanoparticles (AuNPs) outside with 3-aminopropyltriethoxysilane (APTES) as a spacer and glue, denoted as UR@APTES@AuNPs.

**Results:**

The developed LFIA enabled dual-mode PVS detection within 15 min with negligible cross-interference from Potato virus X and Potato virus Y. The platform achieved a limit of detection (LOD) of 31.2 pg/mL for the colorimetric mode and 7.6 pg/mL for the fluorescent mode, over one order of magnitude lower than the conventional AuNPs-based LFIA (LOD = 462.4 pg/mL). Furthermore, PVS in field-collected potato leaves was successfully detected by the new LFIA platform.

**Discussion:**

The dual-mode LFIA demonstrated a rapid, cost-effective, and highly sensitive on-site screening of PVS in field samples, highlighting its great potential in practical application. This platform can be easily adapted to the detection of other pathogenic viruses by using the corresponding specific antibodies.

## 1 Introduction

Potato virus S (PVS) is one of the most dominant viruses infecting Solanaceae plants, especially potato crops. It can be widely transmitted through a variety of ways, including direct contact, insects, infected seed potatoes, and soil (Ospankulova et al., 2023). Upon infection, the virus integrates into the host's cellular environment and persists for the whole lifespan of the plant (Wang et al., 2016; Gong et al., 2019). Generally, PVS infection causes potato yield losses of 3%−20%, and even up to 30% in certain European regions. Coinfection of PVS with other viruses, including potato virus X (PVX) and potato virus M (PVM), can further aggravate yield losses to 40%−75% (Petrov et al., 2023). Since there is currently no effective treatment for infected plants, accurate and efficient viral screening of seed potatoes and field-grown plants is critical for seed potato quality control and inhibiting spread of viral diseases. Therefore, fast, sensitive, and reliable methods are urgently desirable for on-site detection of PVS and other prevalent potato viruses in agricultural production.

Currently, PVS detection primarily relies on enzyme-linked immunosorbent assay (ELISA), polymerase chain reaction (PCR), and quantitative reverse transcription-PCR (qRT-PCR) (Zhang et al., 2016; Onozuka et al., 2021; Tessema et al., 2024). Although these methods offer high analytical sensitivity under controlled laboratory conditions, they are constrained by cumbersome procedures, long processing times (4–8 h), dependence on sophisticated equipment and trained personnel (Song et al., 2017; Yu et al., 2022, 2026). Colloidal gold-based lateral flow immunoassay (LFIA) strip has emerged as a leading candidate due to its operational simplicity, low unit cost, and potential for multiplex detection (Yu et al., 2018). However, the conventional colloidal gold-based LFIA is hindered by two major limitations: insufficient sensitivity with a typical limit of detection (LOD) at ng/mL and poor reliability due to a single-mode readout (Zhang et al., 2021). Recent works focused on the development of nanocomposites by integration of different signals into one probe, such as hybrid nanostructures combining PBNPs@mAbs, PPyNPs@metal-phenolic materials (PMNPs), and MIL-101(FeCu)@AuNPs (Yu et al., 2024; Yuan et al., 2025; Lin et al., 2026). Through these efforts, the LODs were improved to the sub-ng/mL range while increasing the detection reliability based on the dual-mode readout.

Metal-organic frameworks (MOFs), formed by the assembly of metal nodes and organic linkers, have a high loading capacity of target molecules due to their porosity (Gao et al., 2022; Yu et al., 2023, 2025). MOFs have demonstrated as a versatile platform for various applications, such as drug delivery and bio-/chemical sensing (Jiang et al., 2025; Tiwari et al., 2026). Among various MOFs, zirconium-based UiO-66 possesses high porosity, excellent structural stability, and favorable aqueous dispersibility, showing promising applicability in diverse biological fields such as drug delivery, biosensing, and cancer therapy (Dastneshan et al., 2023). Guest molecules such as fluorophores can be efficiently loaded into the porous UiO-66, forming a hybrid fluorescent probe with bright emission to improve the performance in bioanalysis. Despite these advantages, it is still a challenge to effectively immobilize the antibody onto the UiO-66 surface to prepare an immuno-probe for the LFIA strip. In conventional AuNPs-based LFIAs, antibodies are easily immobilized on AuNPs through simple physical adsorption. Thus, the integration of fluorophore-loaded UiO-66 and AuNPs provides an attractive way to construct a novel immune-probe for high-performance LFIAs.

**Scheme 1:**
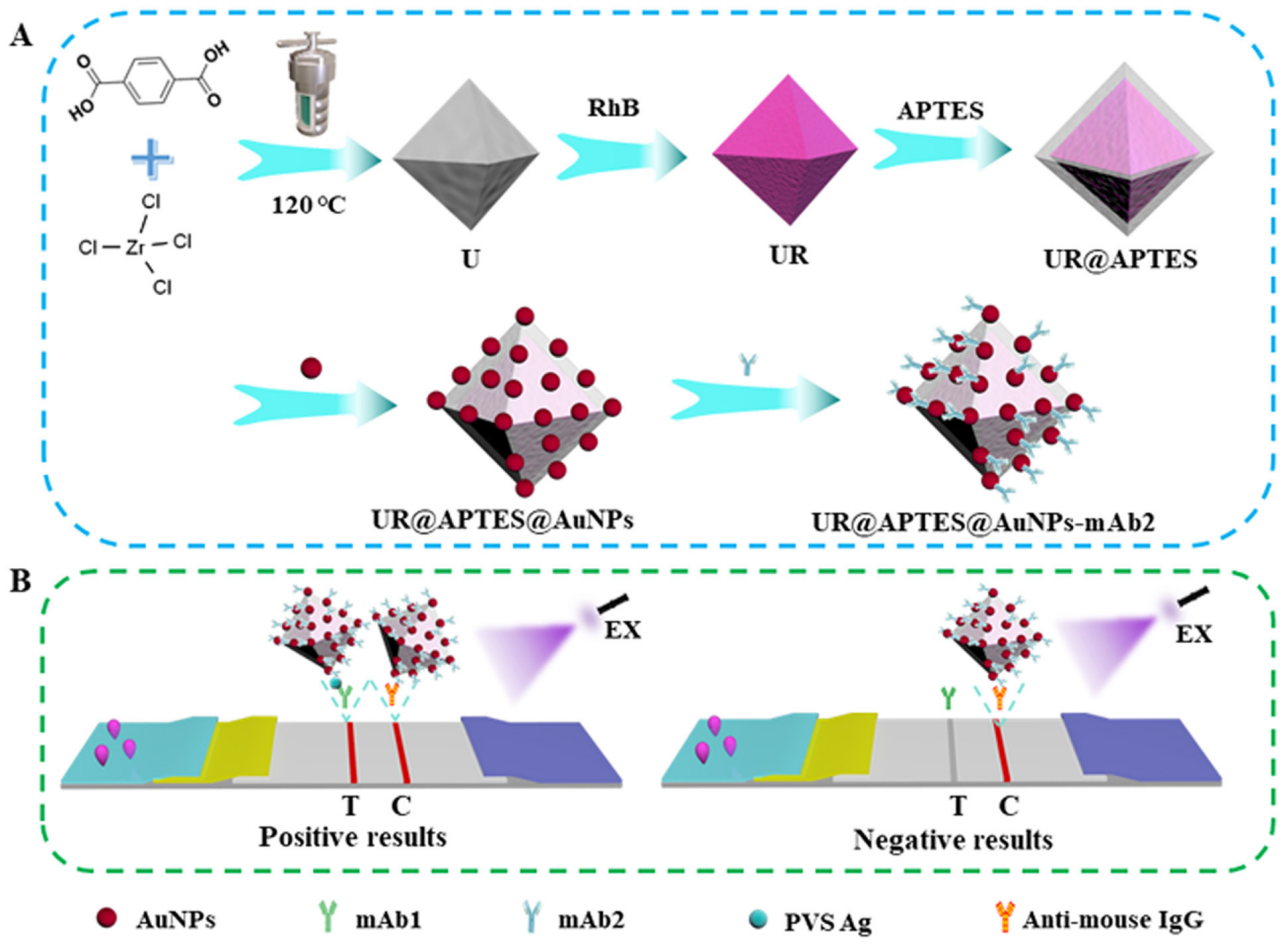
Schematic illustration of **(A)** UR@APTES@AuNPs preparation and **(B)** dual-signal LFIA strip for PVS detection.

Rhodamine B (RhB) has excellent water solubility, high fluorescence efficiency, and superior biocompatibility. It also exhibits stable fluorescence under diverse environmental conditions. Herein, RhB was introduced into UiO-66 by direct mixing to prepare UiO-66@RhB (UR) fluorescent probe. A mild silanization was subsequently implemented using 3-aminopropyltriethoxysilane (APTES), followed by immobilization of gold nanoparticles (AuNPs) on the UR surface via electrostatic adsorption and coordination, resulting in the formation of a core-shell nanocomposite designated as UR@APTES@AuNPs. Scheme 1A depicts the stepwise synthesis of UR@APTES@AuNPs. This silanization layer not only acts as a glue for AuNPs adsorption, but also serves as a spacer to inhibit AuNPs-induced fluorescence quenching of RhB. The dual-signal UR@APTES@AuNPs probe carries multiple AuNPs and numerous RhB molecules, significantly enhancing the colorimetric and fluorescent signal intensity for a highly sensitive PVS assay. Moreover, this modular strategy can be readily extended to diverse combinations of organic chromophores and noble-metal nanoparticles, opening a new avenue for simultaneous multi-target detection and on-site rapid screening in complex samples.

## 2 Materials and methods

### 2.1 Reagents and instruments

Sodium citrate, hydrogen tetrachloroaurate (III) trihydrate, sodium hydroxide, bovine serum albumin (BSA), sucrose, RhB, tween-20, 1-ethyl-3-(3-dimethylaminopropyl)carbodiimide (EDC), terephthalic acid (TPA), zirconium (IV) chloride (ZrCl_4_), N, N-Dimethylformamide (DMF), N-hydroxysuccinimide (NHS), acetic acid, and ethanol were purchased from Aladdin (Shanghai, China). APTES was purchased from Macklin (Shanghai, China). Phosphate-buffered saline (PBS, pH 7.4) was obtained from Beijing Dingguo Changsheng Biotechnology Co., Ltd. (Beijing, China). Nitrocellulose (NC) membranes, glass fiber, absorbent paper, and polyvinyl chloride (PVC) sheets were purchased from Shanghai Jinbiao Biotechnology Co., Ltd. (Shanghai, China). Monoclonal anti-PVS1 (mAb1, immobilized on NC membrane) and monoclonal anti-PVS2 (mAb2, conjugated on probe) were kindly provided by Professor Jianxiang Wu, Zhejiang University. PVS (agdia) antigen was ordered from Beijing Zhongjian Baotai Biotechnology Co., Ltd. (Beijing, China). An ELISA kit for PVS quantification was obtained from Shanghai Yuanju Biotechnology Center (Shanghai, China). The morphology and dimensions of the synthesized nanomaterials were characterized using field emission scanning electron microscopy (FESEM, JEOL JSM-7800F). UV-visible absorption spectra were recorded with a UV-Vis spectrophotometer (UV-2550, Shimadzu), and photoluminescence spectra were collected using a fluorescence spectrophotometer (FluoroMax-4, Horiba).

### 2.2 Synthesis of UiO-66

30 mL of DMF and 2 mL of acetic acid were injected into a 50 mL beaker and stirred until a homogeneous solution was obtained 0.160 g of ZrCl4 was then dissolved in the above solution with the assistance of magnetic stirring, followed by the addition of 0.115 g of TPA. The mixture was stirred continuously for more than 30 min to ensure thorough dispersion of the ligand. The resulting solution was transferred to a polytetrafluoroethylene-lined autoclave, sealed, and reacted at 120 °C in an oven for 24 h (Liao et al., 2023). After the system was cooled to room temperature, the product was collected by centrifugation, followed by three times washing with DMF and absolute ethanol, respectively. The resulting precipitate was dried in a vacuum oven at 120 °C for 12 h, achieving a white UiO-66 powder for subsequent experiments.

### 2.3 Preparation of UR

100 mg of dried UiO-66 powder was dispersed in 20 mL of anhydrous DMF, followed by the addition of different amount of RhB under continuous stirring for 2 h at room temperature. The final concentrations are 0.1, 0.5, 1.0, 1.5, 2.0 mg/mL. RhB was loaded into the pores of UiO-66 mainly through π-π stacking and electrostatic interaction. The resulting UR was collected and washed repeatedly with DMF and ethanol by centrifugation until the supernatant became colorless, followed by vacuum-assisted drying at 60 °C for 12 h.

### 2.4 Preparation of UR@APTES@AuNPs

50 mg of UR powder was dispersed in 50 mL of anhydrous toluene in a 100 mL two-neck round-bottom flask equipped with a reflux condenser. The mixture was stirred under reflux at 80 °C for 30 minutes to completely remove residual moisture. 150 μL of APTES (0.3% mass-to-volume ratio) was then introduced via syringe injection, and the reaction was maintained under reflux at 80 °C for an additional 1 h. Upon completion, the mixture was cooled to ambient temperature. The silanized UR product was collected and thoroughly washed with anhydrous ethanol through repeated centrifugation, followed by vacuum-drying at 60 °C for 12 h, yielding an amine-functionalized product UR@APTES.

For AuNPs coating, 20 mg of UR@APTES was dispersed in 40 mL of a freshly synthesized AuNPs (≈5 mg/L). The mixture was stirred at 500 rpm for 12 h at room temperature in the dark, facilitating the adsorption and immobilization of AuNPs onto the UR@APTES surface via —NH2 facilitated electrostatic adsorption and interaction between nitrogen's lone pair electrons and gold surface (Lyu et al., 2023). The probe UR@APTES@AuNPs was obtained and stored at 4 °C for further use.

### 2.5 Preparation of the mAb2-conjugated UR@APTES@AuNPs (UR@APTES@AuNPs-mAb2) probe

50 μL of 0.1 M NaOH was added to the UR@APTES@AuNPs suspension to adjust its pH to approximately 9. 10 μL of mAb2 was introduced into the above suspension, followed by incubation at 37 °C for 2 h to complete the conjugation. Unbound sites were blocked with 1 % BSA at 37 °C for another 30 min to prevent the nonspecific adsorption in the testing steps. Free antibody in the suspension was thoroughly removed by three times centrifugation. Finally, the pellet was resuspended with 100 μL of storage buffer (0.01M PBS containing 10 % sucrose, 0.05 % Tween-20) via a brief sonication to obtain the UR@APTES@AuNPs-mAb2 probe.

### 2.6 LFIA strip construction and PVS detection

The LFIA strip was assembled by laminating a sample pad, conjugate pad, NC membrane, and absorbent pad onto a PVC backing sheet, with each component overlapping the adjacent one by 2 mm (Scheme 1B). The conjugate pad was pre-treated by immersion in a stabilizing buffer (containing 10% sucrose and 0.05% Tween-20) for 30 min. After completely drying at 37 °C for 2 h, the suspension of UR@APTES@AuNPs-mAb2 was uniformly sprayed on the pre-treated conjugate pad, and then dried again at 37 °C. 1 μL of mAb1 and anti-mouse IgG were dispensed onto each NC membrane to form the test (T) and control (C) lines, respectively. After vacuum-drying at room temperature for 2 h, the membrane was blocked with 1 % BSA for another 30 min, followed by vacuum-drying again.

During testing, the sample applied to the sample pad migrates laterally via capillary force. The analyte PVS was recognized by the UR@APTES@AuNPs-mAb2 on the conjugate pad to form the immune complex UR@APTES@AuNPs-mAb2-PVS, which was then captured by the immobilized mAb1 at the T line, yielding a visible red band (primary colorimetric signal) due to the aggregation of UR@APTES@AuNPs. Under handheld UV lamp (365 nm) excitation, intense fluorescence (secondary fluorometric signal) was observed and captured using a digital camera equipped in a mobile phone. The intensities of both signals correlated linearly with the analyte concentration, enabling semi-quantitative or quantitative analysis.

## 3 Results

### 3.1 Characterization of UR@APTES@AuNPs

**Figure 1:**
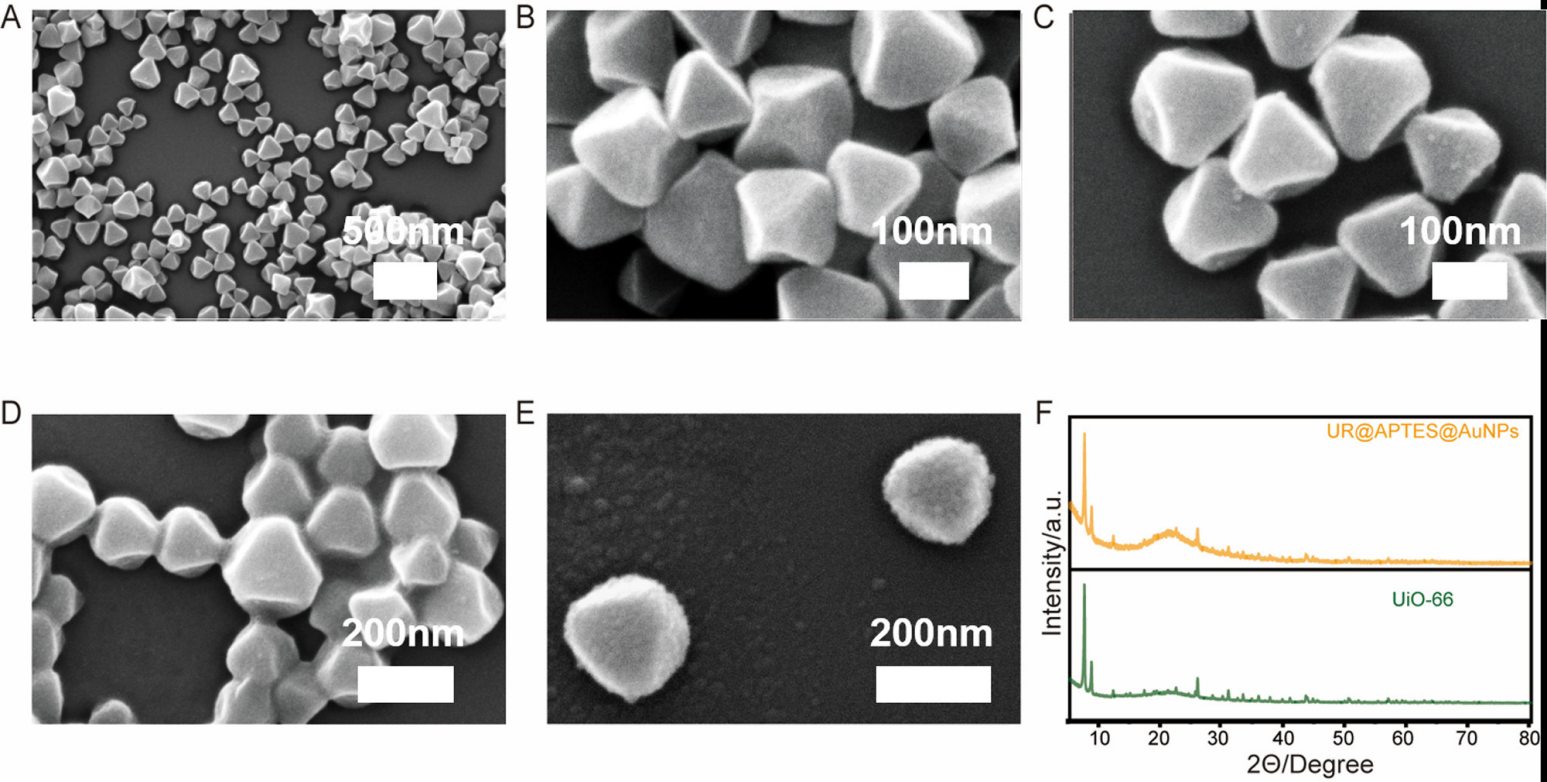
Morphology and phase analysis. FESEM images of **(A, B)** UiO-66, **(C)** UR, **(D)** UR@APTES, **(E)** UR@APTES@AuNPs at different magnifications, and **(F)** XRD pattern of UiO-66 and UR@APTES@AuNPs.

Morphology and size of UiO-66, UR, UR@APTES, and UR@APTES@AuNPs were characterized via FESEM and TEM. As shown in Figures 1A, B, the pristine UiO-66 exhibits a highly regular octahedral structure with a smooth surface and an average edge length of 150 ~ 200 nm. The N_2_ adsorption isotherm of UiO-66 (Supplementary Figure S1) shows a typical Type I profile, which indicates a well-defined microporous structure. Based on the BET result, the specific surface area of UiO-66 was calculated to be 2043.54 m^2^/g. This substantial surface area can provide superior capacity for RhB loading. Figure 1C revealed that the octahedral morphology remains unchanged after RhB encapsulation, underscoring the robust structural integrity of the framework. Subsequent APTES silanization of UR (Figure 1D) also did not make any change to the original octahedral geometry of UiO-66, but yielded a rougher surface. After AuNPs coating, FESEM images (Figure 1E) showed that many spherical AuNPs were decorated on the smooth surface of each UR@APTES nanocomposite, thereby confirming the successful fabrication of UR@APTES@AuNPs. Since each UR@APTES@AuNPs carrier contains many AuNPs, the colorimetric signal will be significantly amplified at the T lines compared to AuNPs alone. These results clearly verified that the octahedral frameworks of UiO-66 were not affected throughout RhB loading, APTES silanization, and AuNPs coating, demonstrating its exceptional stability and capacity to serve as an ideal platform for both colorimetric and fluorescent signal amplification.

The crystallinity of pristine UiO-66 and the core-shell structured UR@APTES@AuNPs was characterized by XRD (Figure 1F). UiO-66 displayed three distinct diffraction peaks at 2θ = 7.36°, 8.52°, and 25.7°, which were assigned to the (111), (200), and (600) crystal planes, respectively. This is consistent with the reported octahedral symmetry. The XRD pattern of UR@APTES@AuNPs was almost identical to that of the parent UiO-66, confirming that neither the RhB encapsulation, APTES silanization, nor the subsequent AuNPs coating altered the intrinsic framework or introduced detectable defects. Notably, no crystalline Au peaks were observed in the XRD pattern, which could be attributed to the low Au content and the nanoscale size. Under these conditions, the scattering contribution from Au was negligible compared to the intense Bragg signals from the UiO-66 framework.

**Figure 2:**
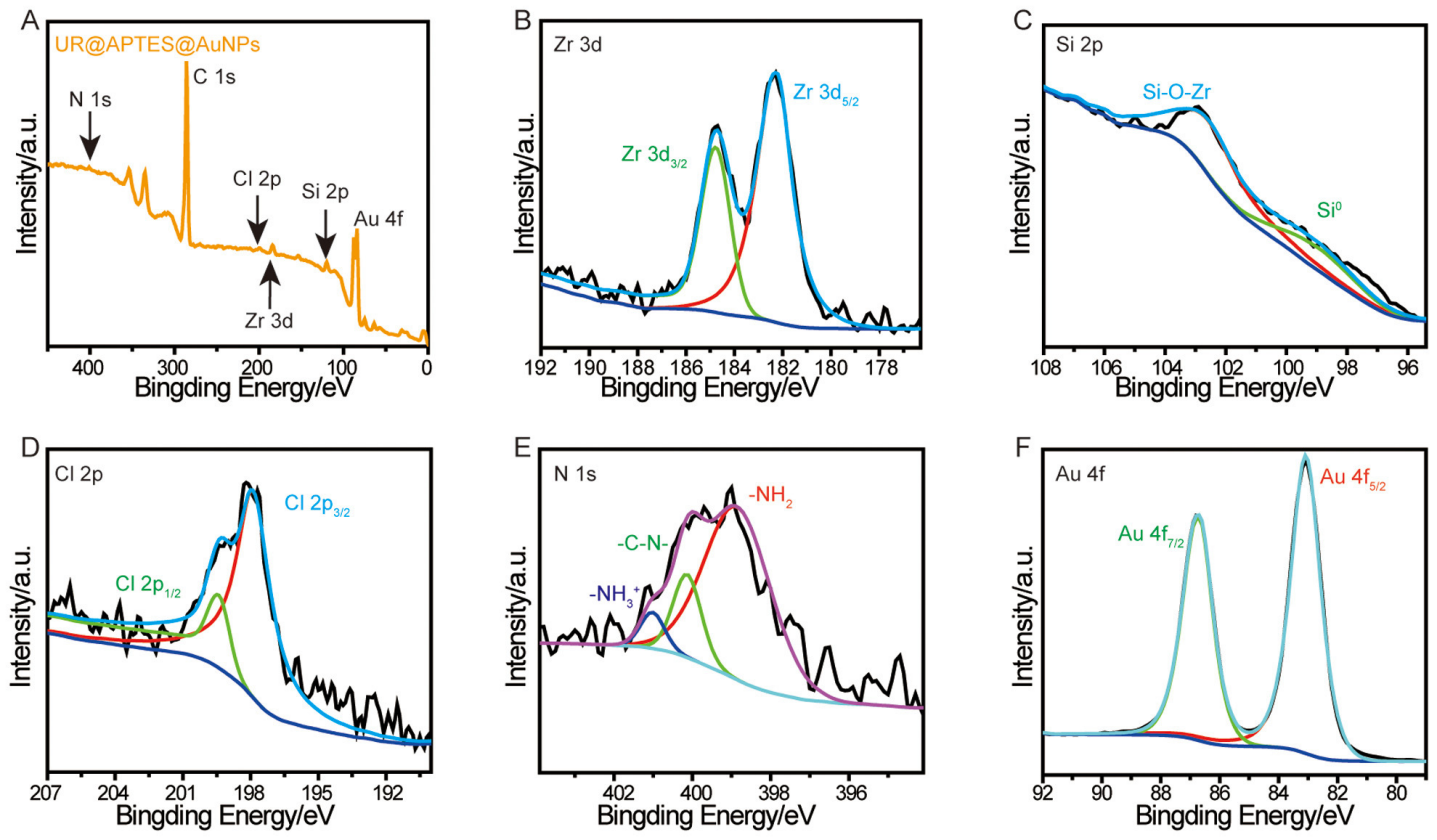
XPS characterization of UR@APTES@AuNPs. **(A)** survey scan and **(B–F)** high-resolution spectra of Zr 3d, Si 2p, Cl 2p, N 1s, and Au 4f, respectively.

Furthermore, X-ray photoelectron spectroscopy (XPS) was employed to systematically investigate the chemical composition and electronic state of UR@APTES@AuNPs. All binding energies were calibrated to the C 1s peak at 284.8 eV. The full survey spectrum in Figure 2A confirmed the presence of C, Cl, N, Si, Au, and Zr. High-resolution scans (Figures 2B–F) sequentially verified the stepwise construction of the composite. Figure 2B presents the high-resolution Zr 3d XPS spectrum of UiO-66. The characteristic doublet with binding energies of 182.2 eV for Zr 3d_5/2_ and 184.8 eV for Zr 3d_3/2_ (ΔBE = 2.6 eV) is consistent with reported values for Zr^4+^, confirming the integrity of the framework's primary building units (Yang et al., 2023a). A noticeable shift of these peaks to lower binding energies was observed, which suggested an increase in electron density around the Zr atoms. This phenomenon provided strong evidence for the successful formation of Si-O-Zr coordination bonds between UiO-66 and APTES. As shown in Figure 2C, the Si 2p spectrum of the UR@APTES@AuNPs composite displayed a weak peak at 99.0 eV and a strong broad peak at 102.5 eV. These binding energies were assigned to elemental silicon (Si^0^) and the Si-O-Zr bond, respectively (Mahajan and Jeevanandam, 2021). The presence of the Si^0^ signal may originate from a silicon wafer substrate. The characteristic Si-O-Zr bond confirmed the successful covalent attachment of APTES to UiO-66 and suggested the formation of a siloxane cross-linked network.

The characteristic Cl 2p XPS peaks in Figure 2D were clearly observed at 197.8 eV and 199.4 eV, corresponding to the Cl 2p_3/2_ and Cl 2p_1/2_ spin-orbit doublet, respectively. The peak separation of 1.6 eV and the area ratio of 2:1 confirmed the successful loading of RhB into UiO-66 (Pratap et al., 2024; Huang et al., 2025). The high-resolution N 1s XPS spectrum shown in Figure 2E could be deconvoluted into three characteristic peaks, clearly revealing the chemical states of APTES on the UiO-66 surface. The peak at a binding energy of 398.8 eV was assigned to the free amino group (—NH_2_) at the terminus of the APTES propyl chain, while the peak at 400.2 eV was attributed to the C—N bond within its structure. Most critically, the characteristic peak observed at 401.0 eV unequivocally corresponded to the protonated ammonium group (—${\text{NH}}_{3}^{+}$), which was highly likely due to an interaction between the —NH_2_ and the Brønsted acid sites on the zirconium clusters of UiO-66 or with protons from the environment (Zhao et al., 2020). These results not only confirmed the successful grafting of APTES onto the UiO-66 surface but also indicated that the positively charged ammonium groups formed on the surface provided active sites for the subsequent loading of negatively charged AuNPs via electrostatic adsorption. Finally, the Au 4f spectrum is dominated by a doublet at 83.1 eV (Au 4f_7/2_) and 86.7 eV (Au 4f_5/2_) (ΔE = 3.6 eV), characteristic of metallic gold (Au). Collectively, the XPS data provided a clear, stepwise account of the assembly process: framework preservation, dye encapsulation, APTES silanization, and AuNPs coating.

**Figure 3:**
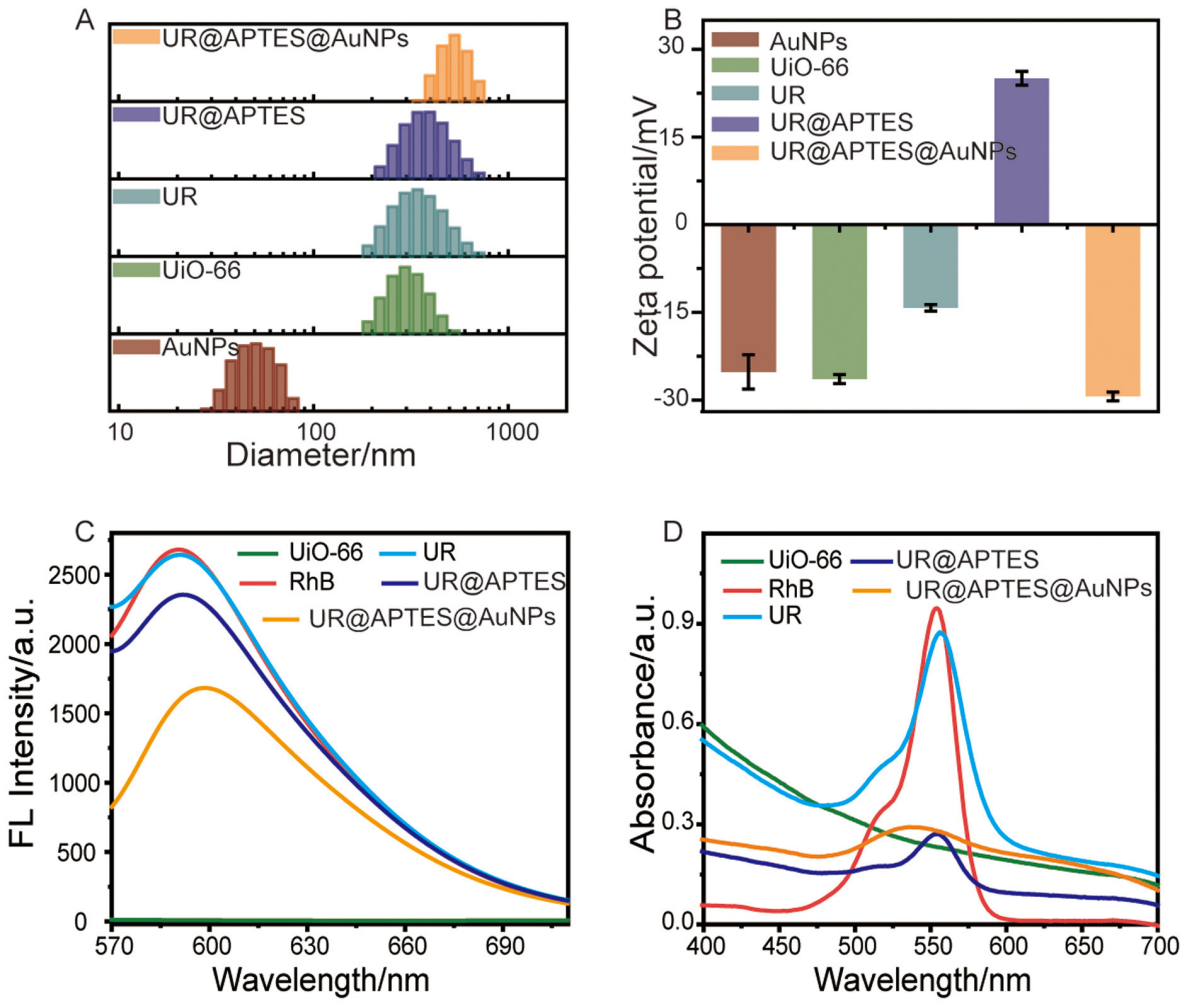
Characterization of UR@APTES@AuNPs. **(A)** DLS analysis, **(B)** Zeta potential of AuNPs, UiO-66, UR, UR@APTES, UR@APTES@AuNPs, **(C)** Fluorescence emission spectra under 550 nm excitation, and **(D)** UV–Vis absorption spectra of UiO-66, RhB, UR, UR@APTES, and UR@APTES@AuNPs.

Dynamic light scattering (DLS) and zeta potential analyses were employed to track the stepwise products during the synthesis of the UR@APTES@AuNPs. Figure 3A presents the hydrodynamic diameters of AuNPs, UiO-66, UR, UR@APTES, and UR@APTES@AuNPs, revealing an increase from 298.11 nm for UR to 520.31 nm for UR@APTES@AuNPs. As shown in Figure 3B, the pristine UiO-66 has a zeta potential of −25.8 mV due to the Zr—O groups on its surface. Since RhB is a cationic dye in neutral aqueous solution, the zeta potential was decreased from −25.8 mV to −14.2 mV after RhB encapsulation in inner pores or adsorption on the surface. The subsequent APTES silanization introduced a dense siloxane layer rich in primary amine, which was converted to —${\text{NH}}_{3}^{+}$ cation upon protonation, resulting in the zeta potential reversal to + 25.0 mV. Finally, citrate-stabilized AuNPs (ζ = −25.2 mV) were coated on the UR@APTES surface via electrostatic force or interaction between the lone electron pair of the nitrogen and the surface Au^0^ atom. The excess citrate ligands on AuNPs introduced a continuous anionic coating, resulting in a final zeta potential of −29.4 mV. The consistent and stepwise changes in both size and zeta potential provide clear evidence for the successful encapsulation of RhB, APTES silanization, and AuNPs immobilization, confirming the successful construction of the dual-signal probe UR@APTES@AuNPs.

Fluorescence and UV-Vis absorbance spectra also provided unequivocal evidence for the successful stepwise fabrication of the UR@APTES@AuNPs probe. As shown in Figure 3C, upon 550 nm excitation, pristine UiO-66 exhibited no emission at a range of 570–700 nm, whereas RhB, UR, UR@APTES, and UR@APTES@AuNPs all showed a strong emission peak at 595 nm, confirming the preservation of RhB fluorescence after each step. However, APTES silanization induced a 5.6 nm bathochromic shift concomitant with a slight intensity attenuation. Meanwhile, the UV-Vis absorption spectra in Figure 3D show that UiO-66 alone has no distinct features in the visible region, while all RhB-containing composites exhibit a characteristic π → π^*^ absorbance at 554 nm. Subsequent amination and AuNPs coating lead to a gradual decrease in absorbance intensity, which was possibly attributed to the high-refractive-index siloxane layer and surface plasmon interactions from AuNPs. The red shift and attenuation observed in both fluorescence and absorbance confirmed the stepwise preparation and the dual-signal characteristics of the UR@APTES@AuNPs probe.

### 3.2 Optimization of experimental parameters

Like the preparation of immune colloidal gold in conventional LFIA, monoclonal anti-PVS antibody mAb2 was simply and efficiently conjugated on the surface of UR@APTES@AuNPs. Dual-signal LFIA was developed based on the UR@APTES@AuNPs-mAb2 probe according to the steps described in Section 2.6. To achieve the best performance, a systematic single-factor optimization was executed for RhB concentration, APTES silanization time, mAb2 concentration, pH, and testing incubation time. Since RhB is a typical aggregation-caused quenching dye, the loading density of RhB is crucial for the fluorescence performance of the probe. As shown in Supplementary Figure S2, as RhB concentration increased from 0.1 mg/mL to 2.5 mg/mL, the emission intensities increased first and then decreased, and the highest peak value was observed at 1 mg/mL. The quenching effect is attributed to the enhanced intermolecular interactions and aggregation of RhB at high concentrations. Therefore, 1.0 mg/mL of RhB was selected as the optimal concentration in subsequent experiments.

**Figure 4:**
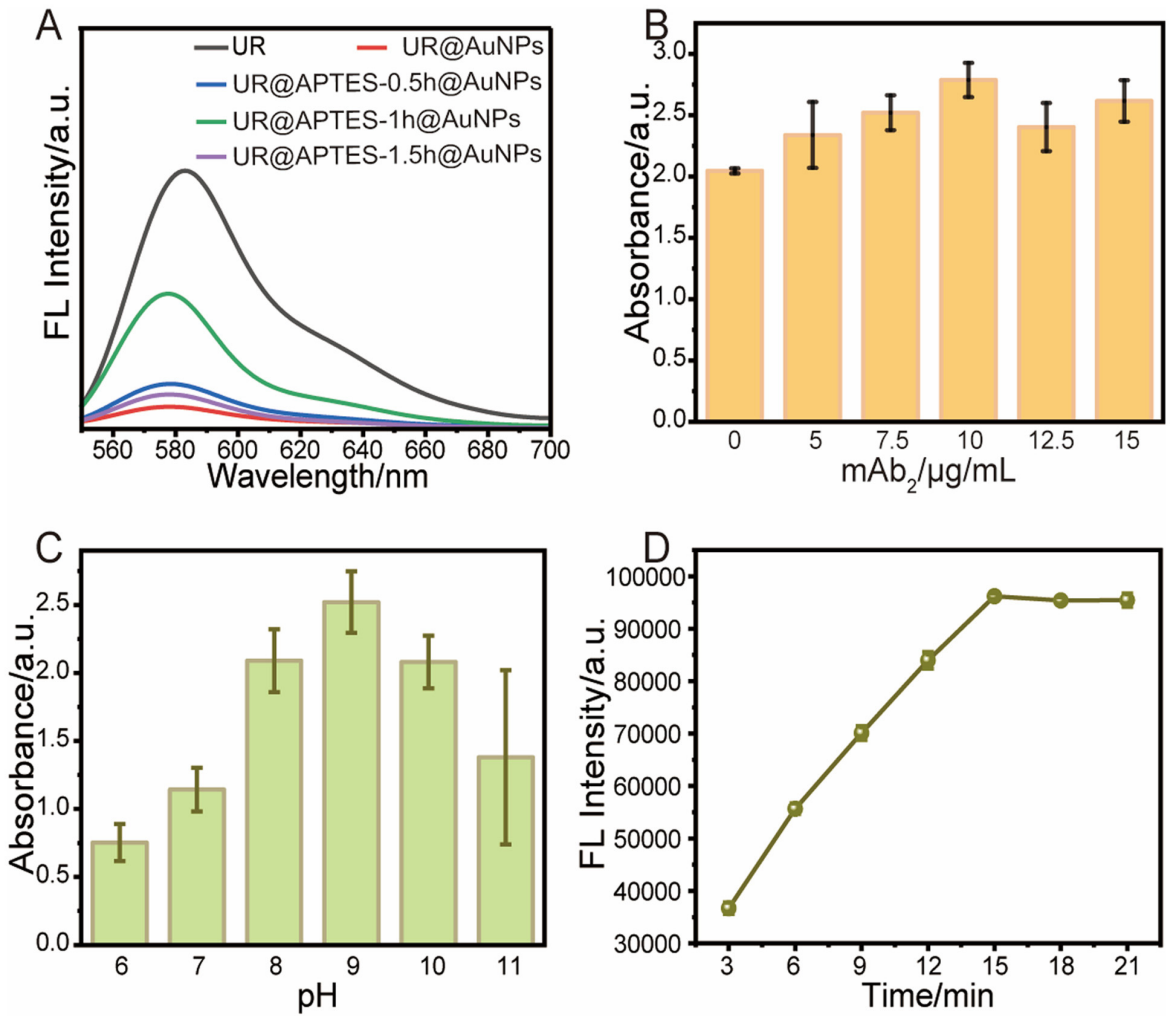
Optimization of experimental parameters. **(A)** The silanization time of APTES, **(B)** Optimization of mAb2 concentration, **(C)** pH in UR@APTES@AuNPs-mAb2 immune probe preparation, and **(D)** incubation time for LFIA.

Direct loading of AuNPs on the UR surface resulted in fluorescence quenching of RhB (Figure 4A). Before AuNPs loading, silica layers with different thicknesses were decorated on UR by varying the silanization time of APTES (0.5, 1, and 1.5 h). The results in Figure 4A indicated that the fluorescence intensity initially increased and then decreased with the elongation of silanization time, reaching a maximum fluorescence emission at 1 h. The optimal silanization time was determined to be 1 h in this system.

Antibody concentration is a critical factor affecting immunoassay performance. Different amounts of mAb2 were mixed with the UR@APTES@AuNPs solution at pH 9, resulting in a variety of final concentrations at 5, 7.5, 10, 12.5, and 15 μg/mL. PBS without mAb2 was utilized as a negative control. Absorbance at 490 nm was measured by a microplate reader. As shown in Figure 4B, the absorbance gradually increased with the increase of mAb2 concentration and reached the maximum at 10 μg/mL. The result indicated that the highest mAb2 conjugation efficiency was achieved at this concentration. Lower mAb2 concentrations resulted in less conjugation, while higher concentrations led to reagent excess without further benefit. Thus, 10 μg/mL is the optimal one and is chosen for all subsequent conjugation steps. pH plays an important role in protein-AuNPs interaction and conjugation efficiency, which subsequently affects the LFIA performance. The pH of the UR@APTES@AuNPs solution in six independent groups was adjusted to 6, 7, 8, 9, 10, and 11, respectively. The mAb2 was then added to each tube at a final concentration of 10 μg/mL and incubated for a predetermined period. Figure 4C showed that the signals increased along with the rising pH up to 9, while a sharp decrease was observed at pH ≥ 10. Time-dependent fluorescence intensities over 0–21 min were shown in Figure 4D for PVS detection using the UR@APTES@AuNPs-based LFIA. Fluorescence emission increased steadily and reached a plateau at approximately 15 min. Based on these results, the optimal conditions were determined to be: 10 μg/mL mAb2 and pH 9 for mAb2 conjugation with UR@APTES@AuNPs, and 15 min incubation time for PVS testing on the LFIA strip.

### 3.3 Dual-signal PVS detection by the UR@APTES@AuNPs-based LFIA strip

**Figure 5:**
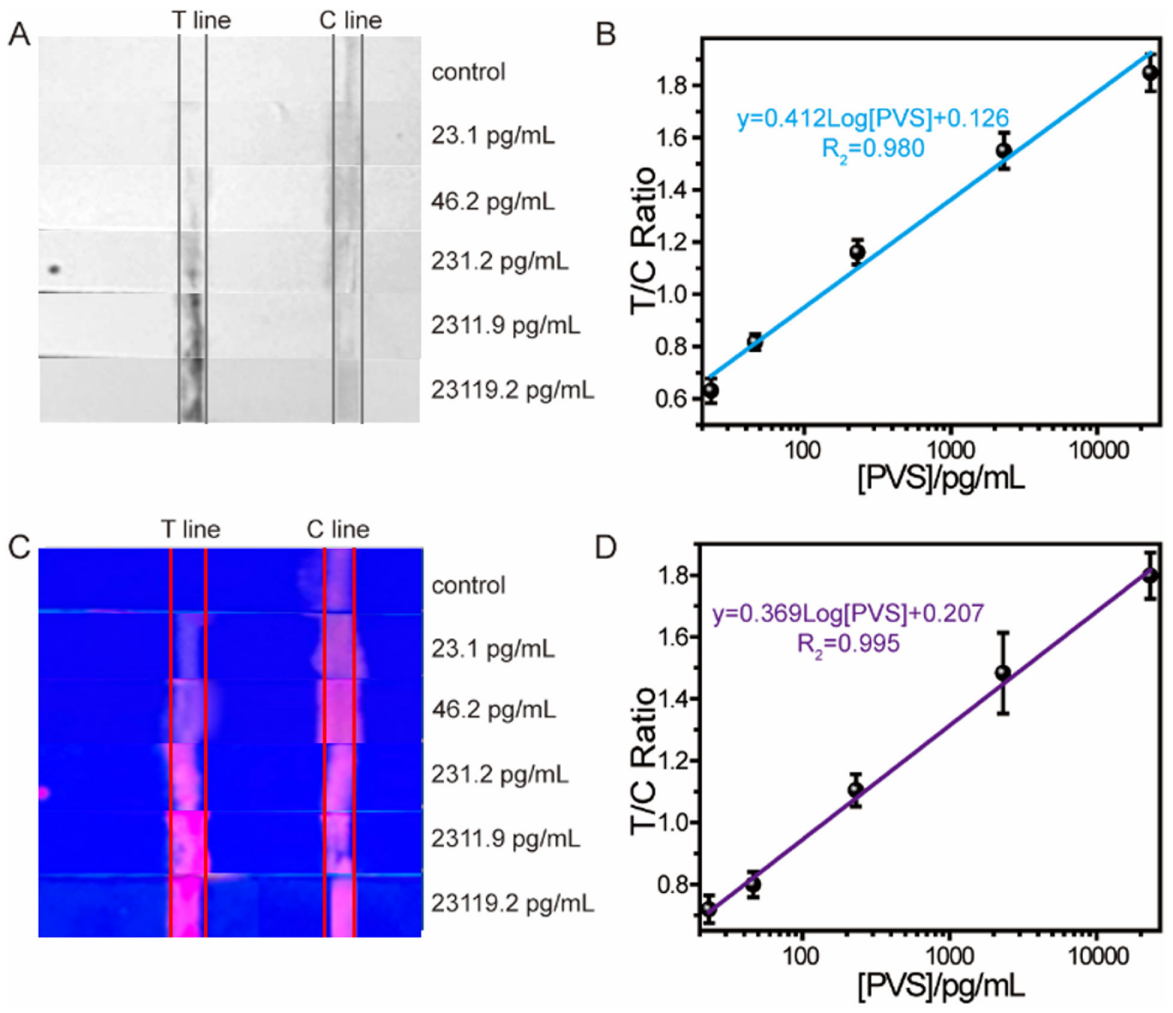
Feasibility assessment of PVS detection. **(A)** bright-field images, **(B)** dose-dependent curve for PVS detection based on colorimetric signal (*n* = 3), **(C)** fluorescent images of LFIA strip, and **(D)** dose-dependent curve for PVS detection based on fluorescent signal (*n* = 3).

The performance of the UR@APTES@AuNPs-based LFIA strip was evaluated by detecting PVS antigens at various concentrations. A stock solution was prepared by dispersing one vial of commercial PVS antigen powder in 2 mL of PBS (pH 7.4). The concentration of the commercial PVS sample was first determined to be 23119.2 pg/mL based on the calibration curve obtained by a PVS virus detection ELISA kit (Supplementary Figure S3). This stock solution was then serially diluted by factors of 1000, 500, 100, 10, and 1, resulting in 5 different concentrations at 23119.2, 2311.9, 231.2, 46.2, and 23.1 pg/mL. These samples were subsequently analyzed using the developed dual-signal LFIA strip. As shown in Figure 5A, under natural light, the colorimetric signal intensity at T line gradually decreased with the decrease of PVS concentration. At the concentration higher than 46.2 pg/mL, the colorimetric signals on the T line were clearly seen by the naked eye. When the concentration was down to 23.1 pg/mL, the T line color could not be distinguished from the blank group. The colorimetric signals of PVS test strips were quantified using Image J software by measuring the gray value intensity of each T line. To minimize variability from strip-to-strip differences, the ratio of background-subtracted T line intensity to background-subtracted C line intensity (T/C ratio) was employed to plot the dose-dependent curve. The resulting calibration curve in Figure 5B showed a good linear relationship between the PVS concentration and the T/C value, with a correlation coefficient (*R*^2^) of 0.980.

Under 365 nm UV excitation (Figure 5C), however, the T line exhibited a distinct pink fluorescence. PVS concentration-dependent fluorescence intensity was observed from LFIA image in Figure 5C. Even at a concentration of 23.1 pg/mL, the fluorescent signal on the T-line could be differentiated from the negative control group, demonstrating the higher sensitivity than the colorimetric mode. Similarly, a good linear relationship between T/C ratio and PVS concentration (Figure 5D) was also obtained in the range of 23.1 to 23119.2 pg/mL (*R*^2^ = 0.995). The LODs of the developed LFIA strip were determined to be 31.2 pg/mL for the colorimetric mode and 7.6 pg/mL for the fluorescent mode based on the formula LOD = 3.3σ/S, where σ is the standard deviation of the blank and S is the slope of the calibration curve. These LODs are more than one order of magnitude lower than that of the traditional colloidal gold-based LFIA (LOD = 462.4 pg/mL) (Supplementary Figure S4). Therefore, the UR@APTES@AuNPs-based LFIA integrates dual-signal outputs to form a robust analytical platform for PVS, significantly extending the dynamic range and improving the detection limit.

The storage stability of the UR@APTES@AuNPs probe is very important to its reliable detection and the shelf-life in practical applications. We further investigated the storage stability of the probe by measuring the hydrodynamic size and fluorescence spectra at days 1, 5, 7, 15, and 30. As shown in Supplementary Figure S5, the particle size on day 30 increased by 3.8% compared to that on day 1. The fluorescence spectra remained a typical RhB emission profile and the peak values only exhibited a slight variation (Supplementary Figure S5). These results clearly indicate good dispersion and storage stability of the probe.

### 3.4 Specificity of the developed LFIA Strip

**Figure 6:**
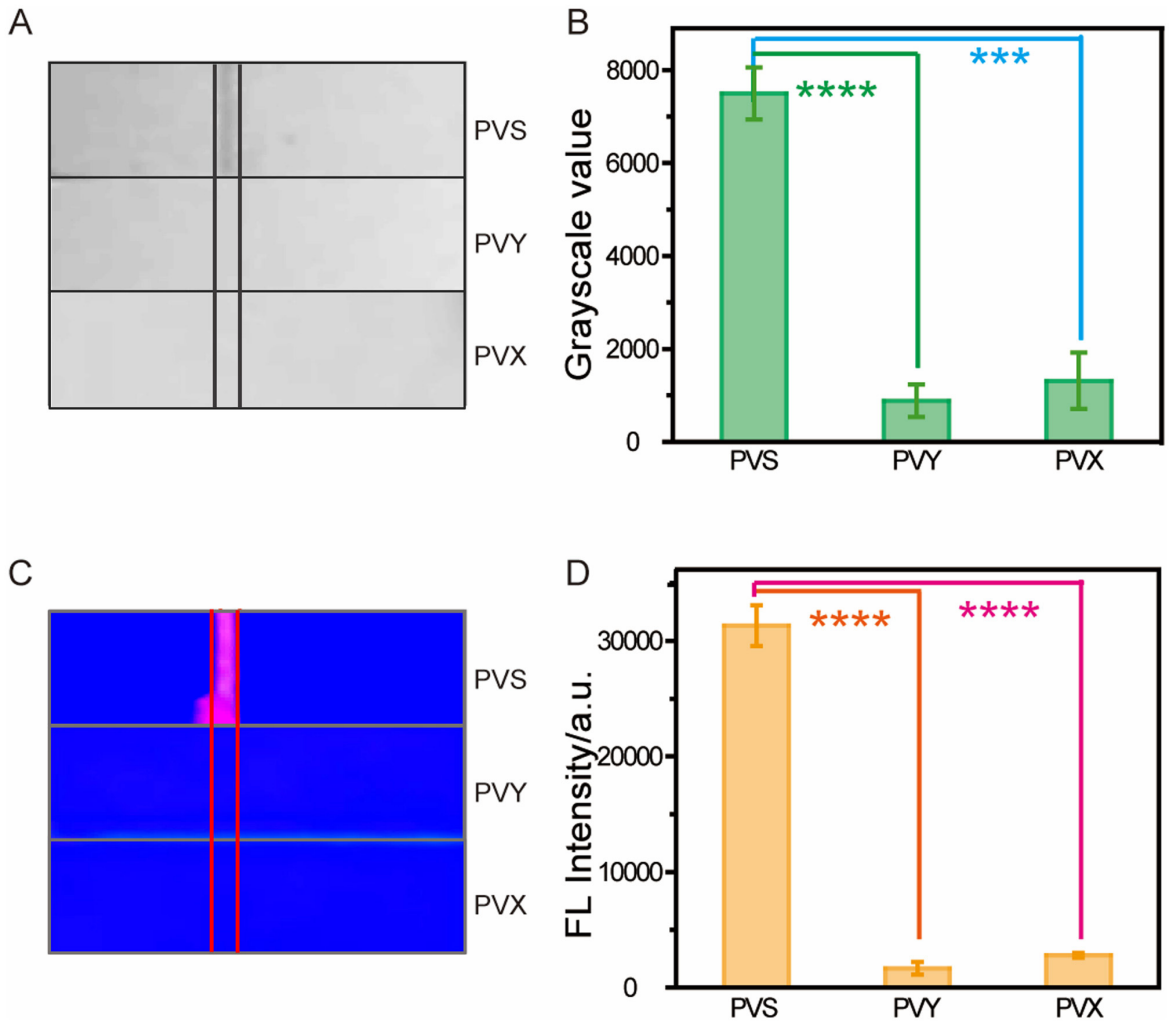
Specificity evaluation for PVS detection. **(A)** bright-field images for PVS, PVY, and PVX detection, **(B)** Quantitative analysis of (A) (*n* = 3, ^***^p < 0.001, ^****^p < 0.0001), **(C)** fluorescent images for PVS, PVY, and PVX detection, and **(D)** Quantitative analysis of (C) (*n* = 3, ^****^p < 0.0001).

Previous studies have indicated that PVX and PVY often coexist with PVS in potato plants, resulting in a potential risk of cross-interference in PVS detection. To evaluate the specificity of the developed LFIA strip, PVS was analyzed in the presence of the potential interferents PVX and PVY, whose concentrations were 10 times that of the targeting analyte PVS. In colorimetric mode, no visible color was developed at the T lines for both PVX and PVY. In contrast, a distinct T line was identified by the naked eye from the PVS group (Figure 6A). The corresponding fluorescent images (Figure 6C) further confirmed these results. There was no detectable fluorescent signal on the T line for either PVX or PVY, whereas a strong fluorescence emission was observed in the PVS group.

Quantitative analyses (Figures 6B, D) demonstrated that in both the colorimetric and fluorescent modes, the developed LFIA showed a good performance even when interferents were present at 10-fold higher concentrations. These findings collectively confirm the high specificity of the LFIA strip for PVS detection.

### 3.5 Practical application for PVS detection in field samples

**Figure 7:**
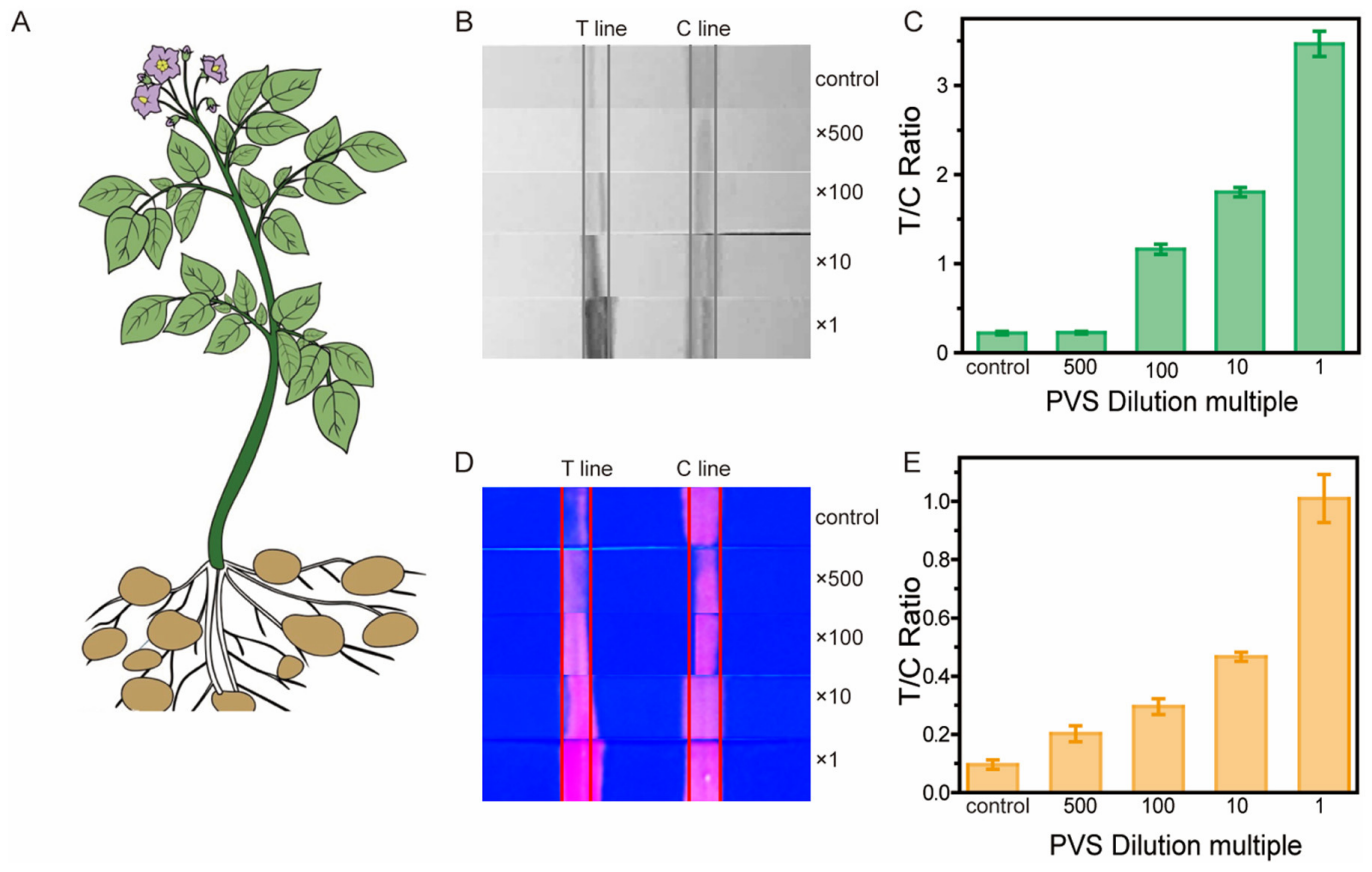
Detection of PVS in field-collected potato leaves. **(A)** picture of potato plant, **(B)** colorimetric and **(D)** fluorescent images for PVS detection in field-collected leaves at different dilution factors (1×, 10×, 100×, and 500×); Quantitative analysis of **(C)** colorimetric and **(E)** fluorescent signals using Image J analysis software (*n* = 3).

To validate the on-site applicability, potato leaves infected with PVS were collected from the field and analyzed with the UR@APTES@AuNPs-based dual-signal LFIA. Figure 7A showed the picture of a potato plant. Infected potato leaves were homogenized in PBS at a 1:10 (w/v) ratio using a mortar and pestle. The resulting homogenate was centrifuged at 2,000 rpm for 10 min, and the supernatant was subsequently passed through a 0.22 μm membrane filter to obtain the PVS-containing sample. After a series of dilutions, the samples were applied to the sample pad of the LFIA strip for detection. Figures 7B, D showed the colorimetric images in bright-field and fluorescent pictures under 365 nm excitation. In both colorimetric and fluorescent modes, the signal intensities decreased progressively with the decrease of concentration. Even at a 500-fold dilution, the signal from the infected specimens was still clearly distinguishable from the PBS negative control, highlighting the superior analytical sensitivity of the fluorescence mode. A linear dose-response relationship was achieved through testing a serial dilution of the field sample (Figures 7C, E). These results demonstrate the great potential of the UR@APTES@AuNPs-based LFIA for fast, highly sensitive, and on-site PVS detection in practical applications.

## 4 Discussion

Labeling probe plays a critical role in LFIA development. In this study, a novel dual-signal LFIA probe, UR@APTES@AuNPs, was developed by efficient encapsulating RhB within the porous UiO-66 and decorating AuNPs onto its surface. The internal RhB generates an intense fluorescent signal, while the external AuNPs enable colorimetric readout and facile antibody conjugation, like conventional colloidal gold-based LFIA. Each UR@APTES@AuNPs probe carries a high payload of RhB molecules and AuNPs, which significantly enhances the colorimetric and fluorescent signal intensity. Compared to conventional AuNPs-based LFIAs, the as-developed LFIA strips exhibit superior detection sensitivity. Moreover, the dual signals of colorimetric and fluorescence can verify each other, further improving the reliability of analytical results. The high-sensitivity dual-signal detection capacity for PVS was further validated in both lab buffer matrices and field-collected PVS-infected leaf samples.

A multilayered SiO_2_-Au core with dual-quantum-dot (QD) shell nanocomposite (denoted as SiO_2_-Au/DQD) has been fabricated to possess prominent colorimetric capability and high fluorescence intensity, enabling the sensitive LFIA detection of monkeypox virus (Yang et al., 2023b). Recently, a multifunctional probe with an Fe_3_O_4_ core loaded with abundant AuNPs and QDs has been exploited for the enhanced colorimetric/fluorescent dual-mode detection of severe acute respiratory syndrome coronavirus 2 (SARS-CoV-2) and monkeypox virus (Wu et al., 2024). Despite their improved analytical performance, these probes require very complicated fabrication procedures. Metal deposition- and bioenzyme/nanozyme-based signal amplification strategies have been demonstrated to significantly improve the LFIA sensitivity (Tian et al., 2019, 2020; Liu et al., 2021; Song et al., 2023). However, deposition reagents or enzyme substrates must be introduced and incubated for different time for signal amplification, thus longer operation time is needed. Additionally, signal output is highly susceptible to reaction conditions (particularly reaction time and temperature), resulting in a poor reproducibility in quantitative detection.

During probe optimization, direct coating of AuNPs onto the UR surface significantly quenched the emission of RhB because of the fluorescence resonance energy transfer (FRET). A silica layer was deposited onto the UR surface as a protective spacer layer to suppress the AuNPs-induced fluorescence quenching (Qin et al., 2018). As expected, the silica shell effectively inhibited the fluorescence quenching effect. The fluorescence recovery was strongly dependent on the separation distance between the AuNPs and the UR carrier. With the distance increases, the energy transfer from fluorophore to AuNPs decreases significantly and an optimal silica shell was obtained with 1 h of APTES silanization. Beyond acting as a separation spacer, the APTES-derived silica shell functionalized the UR surface with positively charged amino groups, facilitating electrostatic adsorption of negatively charged AuNPs. Furthermore, AuNPs were also efficiently anchored via coordination bonding between amine lone-pair electrons and Au(0) atoms on the AuNPs surface (Lyu et al., 2023).

While LFIA offers distinct advantages (low cost, operational simplicity, rapid analysis), it suffers from limited quantitative reliability due to inherent strip-to-strip and batch-to-batch variability. In this work, T and C lines were manually dispensed via pipette owing to laboratory equipment constraints. Despite precise control of antibody loading concentration and volume, notable heterogeneity in T and C line morphology and uniformity was observed on NC membranes. Background-subtracted mean intensities of T and C lines were utilized for quantitative analysis, while T/C ratio, rather than raw T line signal, was employed to minimize inter-strip variability in plotting dose-dependent curves (Zhou et al., 2026).

Despite the favorable analytical performance of the developed LFIA, there are still some limitations for practical applications. First, the synthesis of the UR@APTES@AuNPs probe is quite tedious, multiple steps were involved and several parameters, including the size of UiO-66, the thickness of silica shell, and AuNPs size and concentration could affect the probe's reproducibility from batch to batch. Second, AuNPs aggregation could occur during surface coating onto UR@APTES, resulting in poor sensitivity and reliability. In future study, these parameters should be further optimized, and standardized preparation methods should be developed to obtain UR@APTES@AuNPs probes with good dispersibility, high stability, and minimal batch-to-batch variation. *In-situ* synthesis of AuNPs on UR@APTES surface, instead of coating of pre-synthesized AuNPs, could be an alternative way to prevent AuNPs' aggregation. In addition, with the development of artificial intelligence, the integration of LFIA with smartphone-based imaging and machine learning represents a promising direction to achieve fast and high accuracy quantitative detection (Chen et al., 2026).

## 5 Conclusion

A core-shell structured nanocomposite, UR@APTES@AuNPs, was developed and utilized as a novel probe in an LFIA for highly sensitive dual-mode detection of PVS. The porous UiO-66 framework served as a multifunctional carrier, enabling efficient RhB encapsulation for fluorescent signaling, while supporting the surface decoration of AuNPs to allow direct antibody conjugation and colorimetric detection. This dual-signal platform enabled qualitative on-site screening of PVS via colorimetric readout by naked eye and quantitative analysis through fluorescent mode within 15 min. The developed LFIA strip demonstrated high specificity and achieved LOD of 31.2 pg/mL in colorimetric mode and 7.6 pg/mL in fluorescent mode, representing an improvement of more than one order of magnitude compared to the conventional AuNPs-based LFIA (LOD = 462.4 pg/mL). The practical applicability of the platform was further validated through the successful detection of PVS in field-collected infected potato leaves. In summary, the proposed dual-mode LFIA provides a cost-effective and field-deployable platform for highly sensitive on-site detection of PVS. Moreover, this platform can be easily adapted for the detection of other pathogenic viruses by using the corresponding specific antibodies.

## Data Availability

The original contributions presented in the study are included in the article/Supplementary material, further inquiries can be directed to the corresponding author/s.
