# Supplementary material for: Rhodamine B/gold nanoparticles-coloaded UiO-66 as a novel probe for highly sensitive and dual-mode detection of Potato virus S in field samples
Source: Front Microbiol. 2026 Jun 17;17:1876426. doi: 10.3389/fmicb.2026.1876426 (PMC13319032; doi:10.3389/fmicb.2026.1876426)
Supplement: Supplementary file 1 [file Table_1.docx]

Supplementary Material

# Supplementary Figures





**Supplementary Figure 1.** N_2_ adsorption-desorption isotherms of UiO-66

**Supplementary Figure**

 **2.** Optimization of RhB concentration





**Supplementary Figure 3.** Standard curve for PVS detection by using commercial ELISA kit


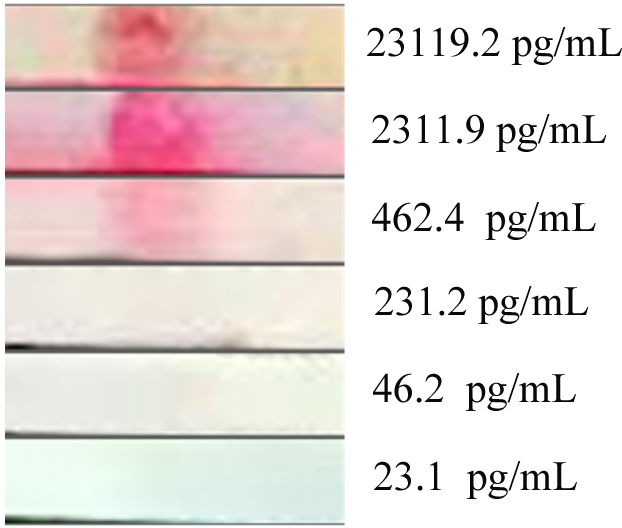


**Supplementary Figure 4.** PVS detection using the traditional colloidal gold-based LFIA


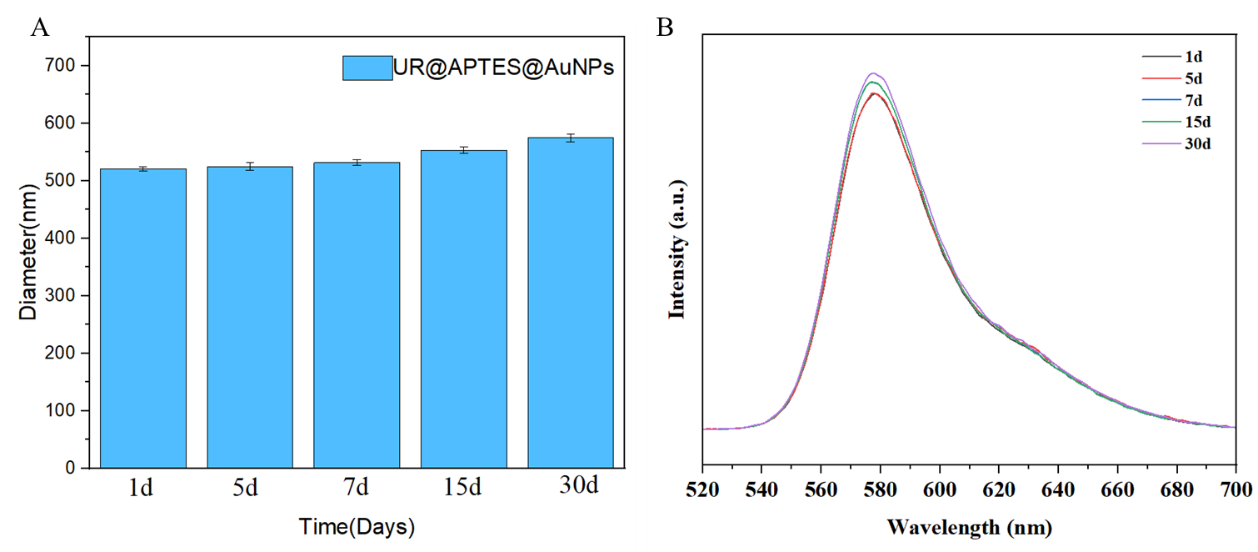


**Supplementary Figure 5.** The storage stability of the developed UR@APTES@AuNPs probe.

(A) Hydrodynamic size and (B) fluorescence spectra of UR@APTES@AuNPs on days 1, 5, 7, 15, and 30.
